# Supplementary material for: A Novel NOX Inhibitor Treatment Attenuates Parkinson’s Disease-Related Pathology in Mouse Models
Source: Int J Mol Sci. 2022 Apr 12;23(8):4262. doi: 10.3390/ijms23084262 (PMC9030373; doi:10.3390/ijms23084262)

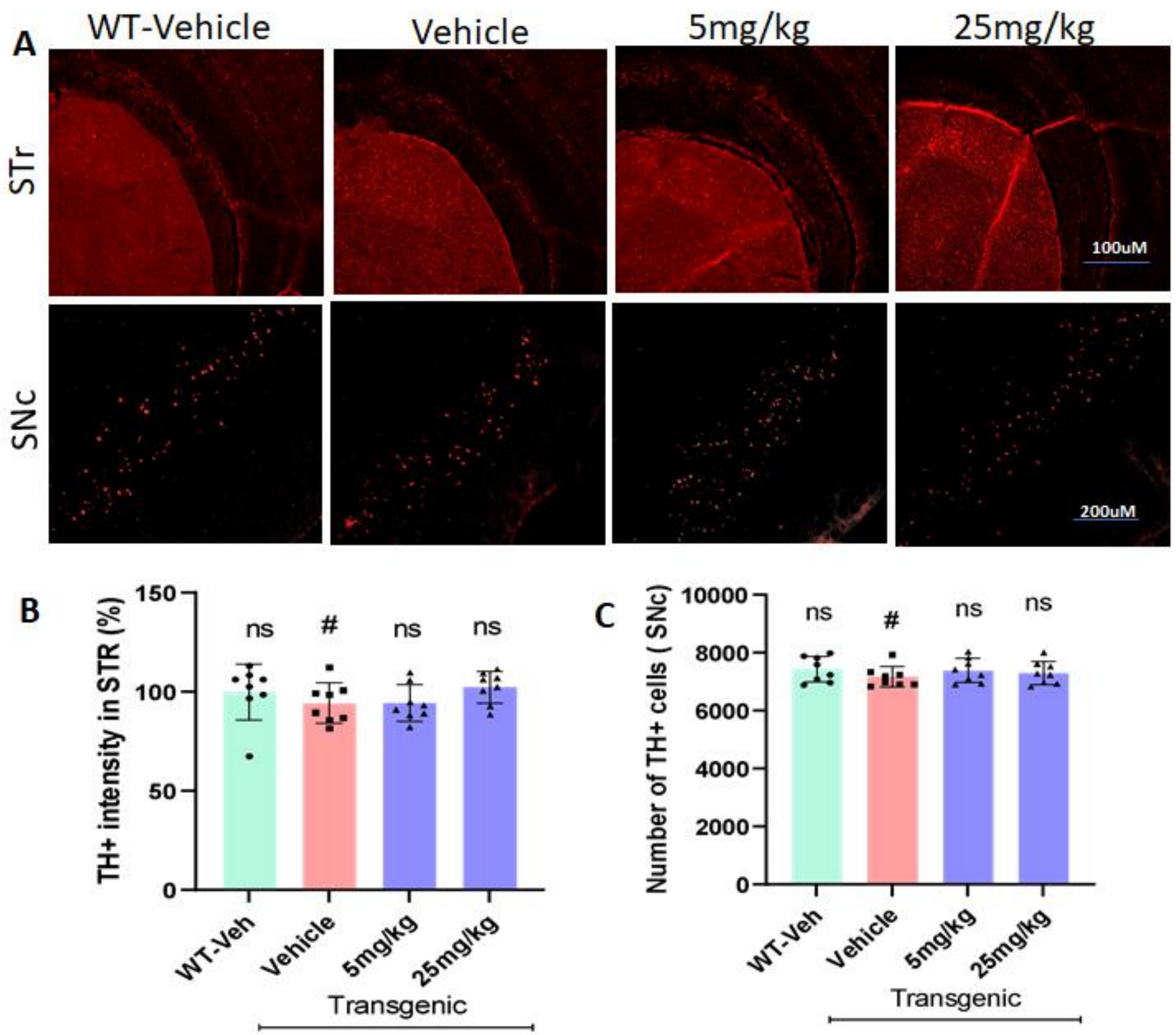

Supplementary Figure S1: The L-61 mice overexpressing human alpha-synuclein did not show any significant changes in the TH intensity for striatum region (A). There was no change in the number of dopaminergic neuron in the SNc region (B). Statistical analysis shows the mice did not have any changes in in the TH+ intensity of number across the groups (B, C).). One-way ANOVA with Dunnett's analysis was used to compare the intensity of TH+ cells in the striatum and TH+ cell number in the SNc, compared to WT-vehicle or tg-vehicle (#) treated mice. ns: not significant.

Supplementary Table S1

Compounds (1 µM)	Concentration	Incubation time	P_e (10^{-6}cm/sec)	BCS code	Method
Progeterone	50µM	4 hrs	29.34	High (CNS+)	
Lidocaine	50µM	4 hrs	20.16	High (CNS+)	UV
Theophylline	50µM	4 hrs	0	Low (CNS-)	
Compound-6	**50µM**	**4 hrs**	**23.94**	**High (CNS+)**	**UV**

Permeability classification		CNS +/− classification	
P_e (10^{-6}cm/sec)	Classification	P_e (10^{-6}cm/sec)	Classification
**> 0.4**	**High**	**> 10**	**+**
**< 0.4**	**Low**	**< 10**	**-**

Supplementary Table S1: Parallel artificial membrane permeability (PAMPA) assay

Uncropped Blot for 7A

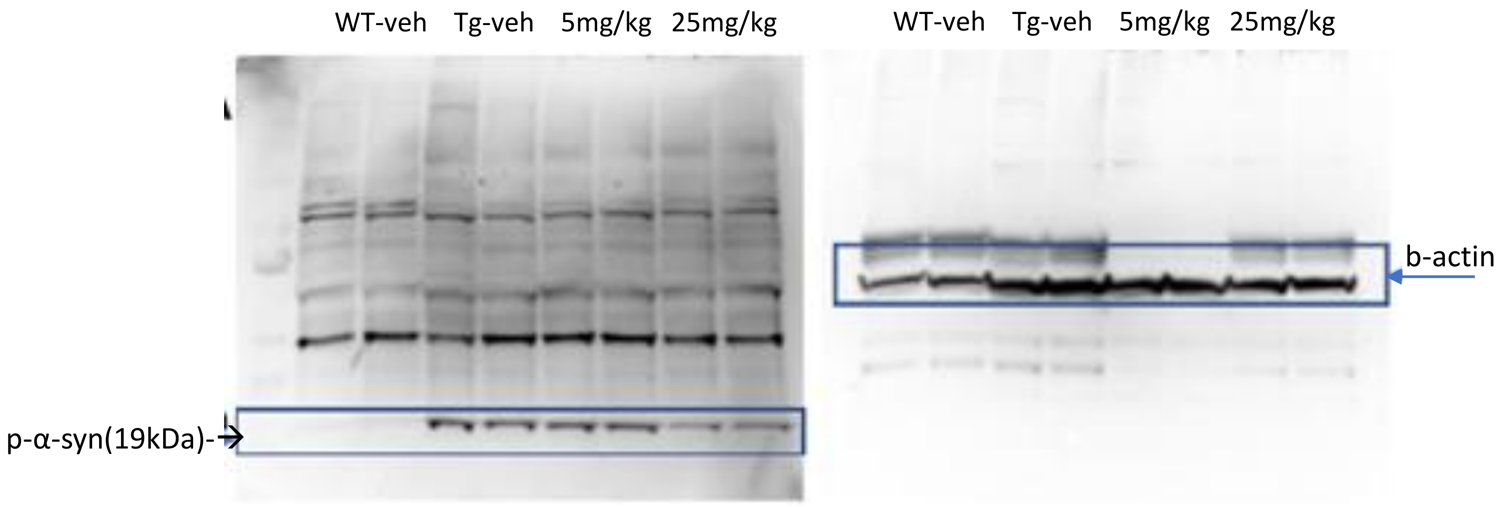

Uncropped Blot for 7B

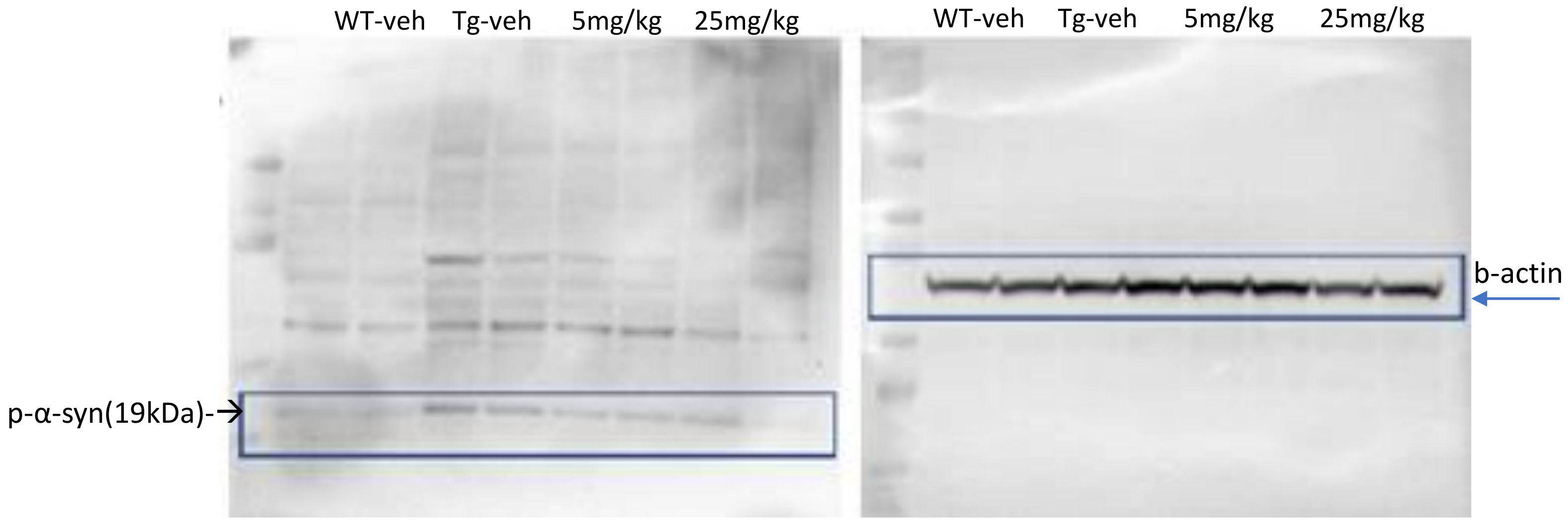

Uncropped Blot for 10A

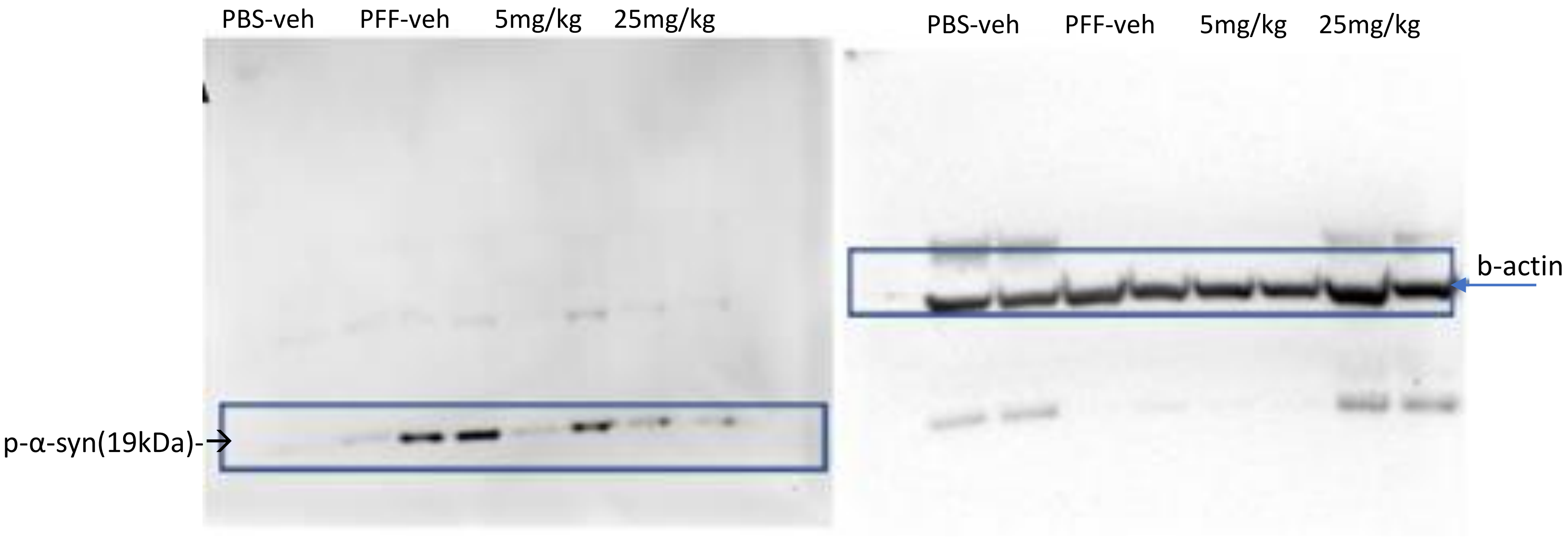

Uncropped Blot for 10B

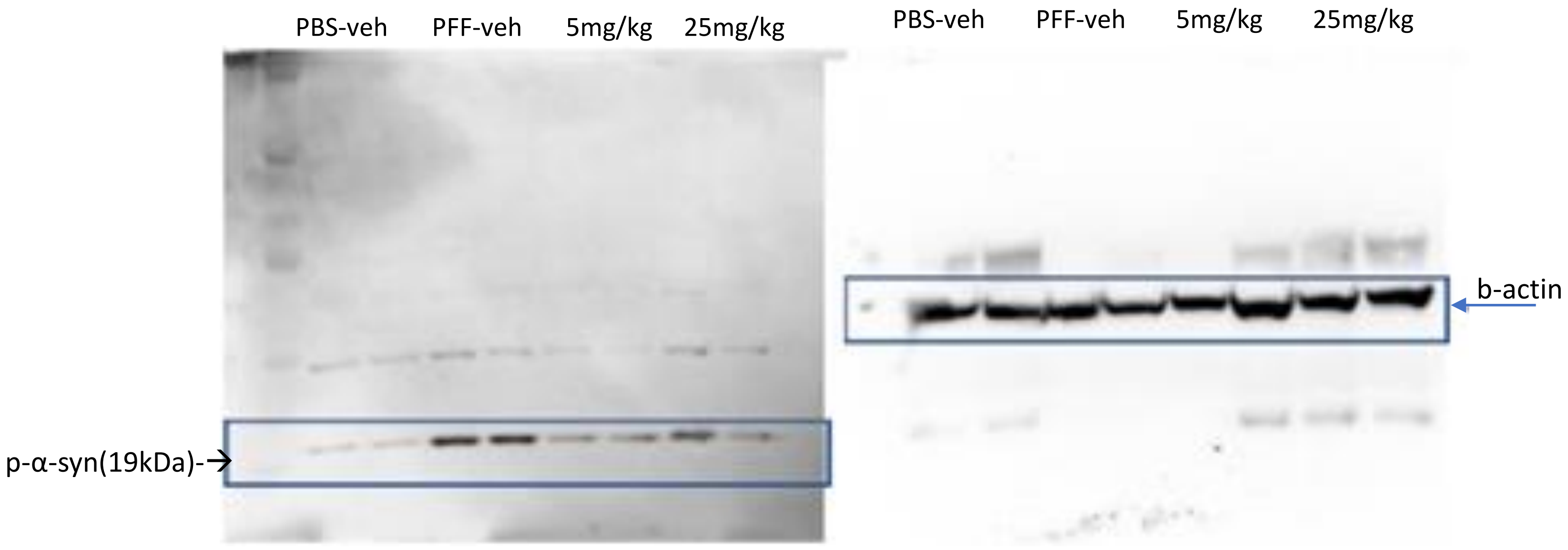

Uncropped images 12A

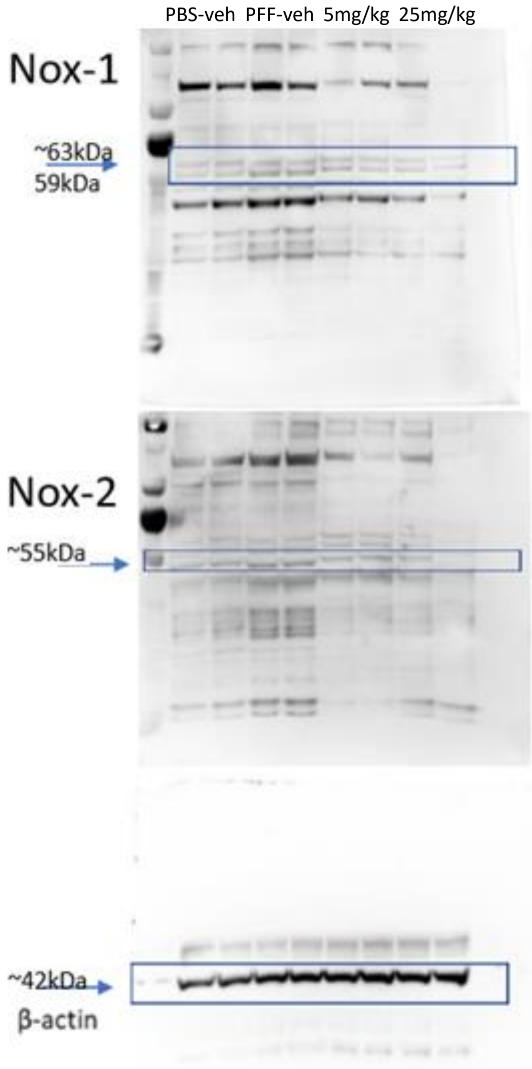

Uncropped images 12B

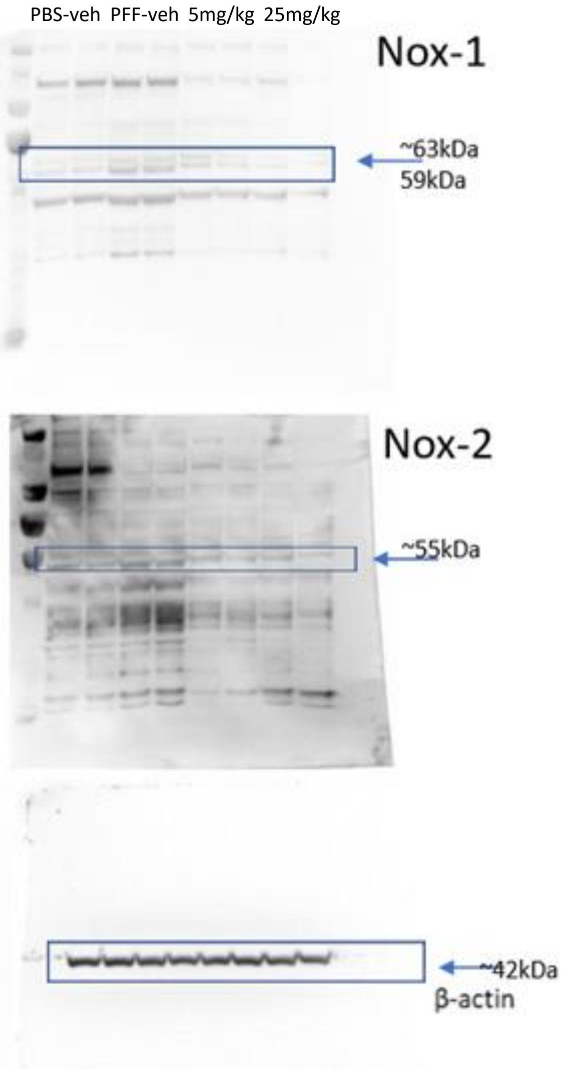

Supplement: Supplementary file 1 [file ijms-23-04262-s001.zip › ijms-1644812-supplementary.pdf]
